# Early Definitive Diagnosis and Management of Incidental Neuroendocrine Tumors Found on Gastrointestinal Endoscopy

**DOI:** 10.7759/cureus.44718

**Published:** 2023-09-05

**Authors:** Kaumudi Somnay, Swapnil Surpur, Prerna Saini, Christopher Gibson, Jean Luo

**Affiliations:** 1 Gastroenterology, NewYork-Presbyterian Queens Hospital, New York City, USA; 2 Internal Medicine, Jawaharlal Nehru Medical College, Belgaum, IND; 3 Internal Medicine, Government Medical College, Patiala, IND; 4 Internal Medicine, The City College of New York, New York City, USA; 5 Pathology, NewYork-Presbyterian Queens Hospital, New York City, USA

**Keywords:** gastrointestinal neuroendocrine tumor, rectal neuroendocrine tumor, eus fna, robotic-assisted surgery, endoscopic ultrasound (eus), incidental gastrointestinal neuroendocrine tumor, gastrointestinal endoscopy, well differentiated neuroendocrine tumor

## Abstract

Neuroendocrine tumors (NETs) are tumors that originate from neuroendocrine cells and can be found throughout the body but are most commonly seen in the gastrointestinal tract, pancreas, and lungs. There is an increase in the diagnosis of NETs due to advances in diagnostic modalities. Although mucosal tumors are easily visualized on upper GI endoscopic imaging, neuroendocrine tumors are often missed due to their deep mucosal origin with normal overlying mucosa.

We first present the case of a 46-year-old woman with anemia and epigastric discomfort who was found to have an incidental submucosal mass in the duodenal bulb on esophagogastroduodenoscopy (EGD), which on endoscopic ultrasound (EUS) with a fine needle biopsy (FNB) showed a neuroendocrine tumor. Imaging with CT, however, failed to detect the presence of the mass in the duodenum. Furthermore, a DOTATATE scan showed only a nonspecific signal near the liver. The patient then underwent an EGD-guided, laparoscopic, robot-assisted transduodenal resection of the tumor, together with the removal of enlarged peritumoral lymph nodes. Pathology showed a well-differentiated neuroendocrine tumor of the duodenal bulb with metastasis to one lymph node, which was confirmed via immunohistochemistry staining.

The second case is of a 51-year-old female who presented with occasional constipation and rectal pain and was found to have a rectal polypoid lesion on her colonoscopy, jumbo biopsies of which revealed a NET. An EUS done for staging and endoscopic mucosal resection (EMR) revealed a grade 1 well-differentiated NET on pathology, which was confirmed by immunohistochemistry staining.

These cases stress the need for timely, definitive diagnosis and intervention. Here, we discuss the clinical features and investigations of neuroendocrine tumors for early diagnosis and management.

## Introduction

Neuroendocrine tumors (NETs) can be found in any organ but are commonly gastroenteropancreatic (GEP), making up roughly 70% of all cases of NETs, with the remainder being of bronchopulmonary and thymic origin. These tumors can remain asymptomatic or present with non-specific symptoms. They can become symptomatic through the release of unregulated hormones or enzyme secretions. Neuroendocrine tumors release substances such as chromogranin A (CgA), serotonin, insulin, glucagon, and others [1]. Symptoms vary depending on the location of the tumor and the specific hormones produced, which can include flushing, diarrhea, pain, wheezing, and weight loss.

Gastroenteropancreatic neuroendocrine tumors (GEP-NETs) represent the second most prevalent digestive cancer. The projected prevalence of NETs in the US population in 2014 was 171,321.7. The small intestine accounted for 30.8%, the rectum 26.3%, the colon 17.6%, the pancreas 12.1%, and the appendix 5.7% of all NET sites throughout the digestive tract. The foregut carcinoid primaries arise from the lung, thymus, and gastric mucosa; the midgut carcinoid primaries arise in the ileum, cecum, or proximal colon; and the hindgut carcinoid primaries arise primarily in the distal colon and rectum. While midgut NETs occur predominantly in white patients, rectal NETs develop more frequently in African American, Asian, and Native American patients. Female patients appear to be more prone to developing NETs of the appendix, or colon, whereas NETs of the small intestine and rectum are more prevalent in male patients. Individuals with a family history of NETs in a first-degree relative have a 3.6-fold increased risk of disease [2]. Neuroendocrine tumors that begin in the gastrointestinal tract are slow-growing tumors that often present in the metastatic phase (40%-80% of patients have metastatic disease when diagnosed). Metastasis of the liver is most common (40%-93%), followed by bone (12%-20%), then lung (8%-10%). Liver metastasis is often followed by a decline in disease-specific survival [3].

The most recent WHO classification, which reclassified the well-differentiated NETs according to their mitotic count, or Ki-67 index, linked them to cellular proliferation. Neuroendocrine tumors are categorized as G1, G2, and G3 [4]. A low-grade tumor, G1, is a tumor with a mitotic rate of less than two mitotic cells per 10 high power fields (HPF); G2 is a moderate-grade tumor that has a mitotic rate of two to 20 per 10 HPF; and G3 is a high-grade tumor that has a mitotic rate greater than 20 per 10 HPF. The Ki-67 proliferation index values for low, moderate, and high grades were less than 3%, 3% to 20%, and greater than 20%, respectively. Poorly differentiated neuroendocrine carcinomas (NECs) are divided into large-cell and small-cell types. Although the prognosis for individuals with G1 NETs is generally favorable, it was reported that the five-year survival rate was between 80% and 90% [5]. Grade 3 NETs indicate metastatic progression, even in tumors that were previously diagnosed clinically as localized. For low-grade, intermediate-grade, and high-grade tumors, the five-year survival rates were 79%, 74%, and 40%, respectively [6].

Imaging modalities such as CT, MRI, or PET can be used to visualize and localize tumors; 68Ga-DOTATATE PET/CT scans are usually used to diagnose individuals with suspected NETs, presenting with clinical symptoms or increased tumor markers, or both, justifying its usage in clinical routine diagnostics [7]. Neuroendocrine tumors can be classified based on their location, size, quality, and whether or not they produce hormones (functional or nonfunctional), or according to the extent and spread of the tumor. The tumor, node, and metastasis (TNM) staging criteria, created by the American Joint Committee on Cancer (AJCC), are used to classify neuroendocrine tumors [8]. Analysis of the Surveillance, Epidemiology, and End Results (SEER) database has validated the relationship between tumor stage and prognosis [9-10]. The treatment approach depends on various factors, such as the size, location, grade, and stage of the tumor and whether it is functional or nonfunctional. Treatment options for NETs include somatostatin analogs, targeted therapy, radiofrequency ablation, radiation therapy, chemotherapy, newer drugs in clinical trials, and, more commonly, surgical, or more recently, endoscopic excision of smaller NETs [11].

According to the TNM classification based on the pathological findings, T0 denotes the absence of a primary tumor; Tis, tumor in situ/dysplasia (size <5 mm); T1, gastric or duodenal tumor invading the lamina propria or submucosa and size <10 mm or pancreatic tumor limited to the pancreas and size <20 mm; T2, gastric or duodenal tumor invading the muscularis propria or subserosa, or size >10 mm, or pancreatic tumor limited to pancreas and size between 20 mm and 40 mm; T3, gastric or duodenal tumor penetrating the serosa, or duodenal tumor infiltrating the pancreas, or pancreatic tumor limited to pancreas and size >40 mm, or pancreatic tumor invading the duodenum or the common bile duct; T4, gastric, duodenal, or pancreatic tumor invading adjacent structures; N0 is the absence of any nodal metastasis, and N1 denotes regional lymph node metastasis; M0 is no distant metastasis; M1 is distant metastasis.

Stage I consists of T1 NETs with slow development. Stage II indicates T2 or T3 tumors, which are greater in size or more invasive, though never with metastases. Stage III refers to either the existence of localized node metastases (stage IIIB) or the invasion of neighboring structures (stage IIIA). Stage IV is always associated with distant metastases [12]. The TNM staging approach examination of 691 patients with NETs in 2000 and 2010 showed five-year survival rates of 100%, 100%, 91%, and 72% for stages I through IV, respectively [13].

A systematic review of incidental findings through diagnostic imaging studies showed that although most incidental abnormal findings are followed up clinically, they are often not clinically confirmed through additional diagnostic testing [14]. In this case series, we diagnosed two patients with NETs that were found as incidental lesions on endoscopy. The presentation of these cases highlights the importance of early and clinical confirmatory diagnosis of abnormal incidental findings, which can improve prognosis after definitive treatment.

## Case presentation

Case one

**Figure 1:**
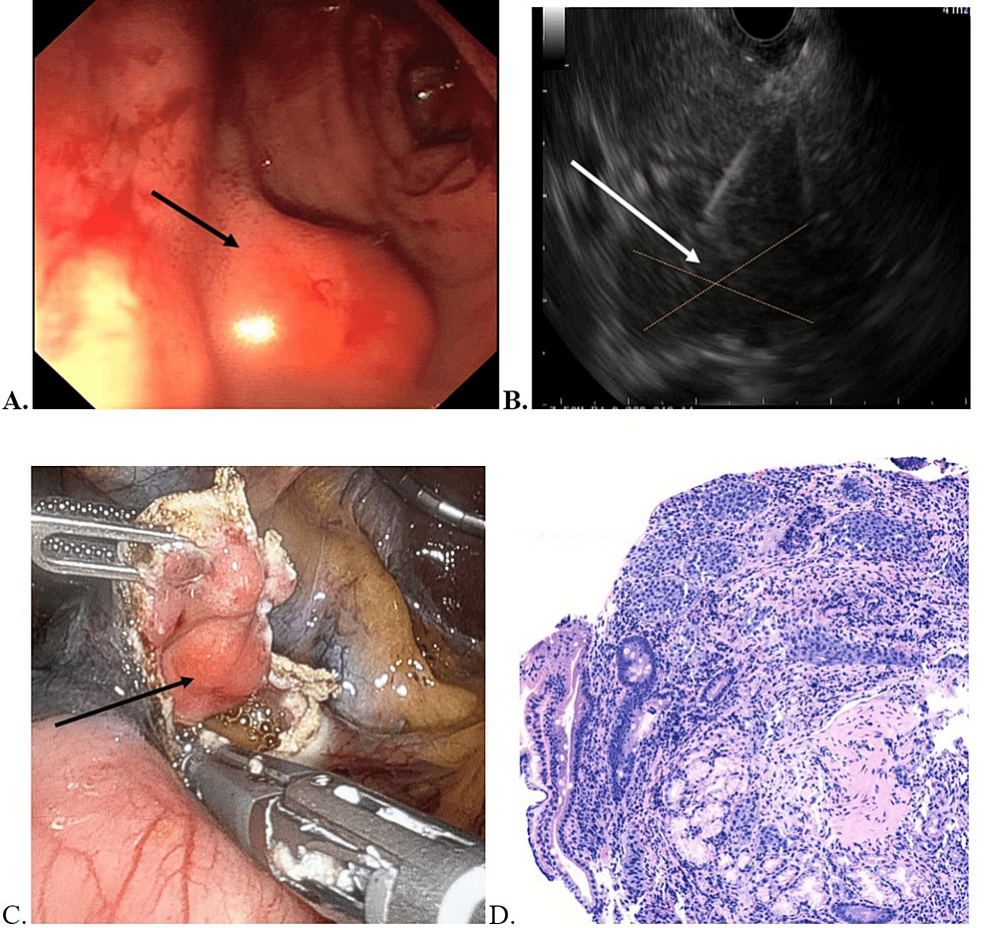
A) Endoscopic visualization of the tumor in the duodenum; B) Image of the EUS-FNA of the mass; C) Gross specimen of the tumor being resected via robotic approach; D) Histological slide of the mass demonstrating characteristic palisading of neuroendocrine cells with fibrovascular septae in between EUS-FNA: endoscopic ultrasound-guided fine needle aspiration

A 46-year-old female was evaluated for long-standing epigastric discomfort and iron-deficiency anemia at our gastroenterology clinic. She complained of burning, non-radiating epigastric pain with an intensity of 6/10 and had presented to the emergency department days prior to her consultation. She denied nausea, vomiting, fever, chills, changes in bowel habits, dysphagia, odynophagia, hematochezia, melena, and weight loss. She complained of heavy menses, for which she was being treated by her gynecologist. Prior to her esophagogastroduodenoscopy (EGD), she was taking amlodipine, ferrous sulfate, and docusate sodium regularly for high blood pressure, anemia, and occasional constipation, respectively. Her surgical history included an appendectomy and two cesarean sections. She had no family history of cancer or any other significant diseases or disorders. She presented with normal vital signs and, upon examination, she had mild conjunctival pallor. There was no associated icterus, cyanosis, or clubbing. Her abdominal examination was normal. All other aspects of her physical examination were unremarkable. Her complete blood count revealed hemoglobin and hematocrit levels of 10.4 g/dL and 33.9%, respectively, with a mean corpuscular volume (MCV) of 89.4 fL and a red cell distribution width (RDW) of 13%. Her WBC, platelet count, and comprehensive metabolic panel were within normal limits. Her iron panel showed iron deficiency anemia, with serum iron levels of 47 μg/dL, total iron binding capacity of 394 μg/dL, ferritin of 8 ng/mL, and transferrin saturation of 12%. She underwent an EGD that showed *Helicobacter pylori* (*H. pylori*)-associated gastritis, focal intestinal metaplasia in the antrum, and a submucosal mass in the duodenal bulb (Figure 1A).

She was treated for *H. pylori* gastritis with a combination antimicrobial therapy. Biopsies of the duodenal bulb mass showed only hyperplastic changes in pathology. She was then scheduled for endoscopic ultrasound (EUS) (Figure 1B) and fine needle aspiration (FNA) or fine needle biopsy (FNB) for evaluation of the submucosal mass. The pathology report of the biopsy and cytology of the mass revealed a well-differentiated neuroendocrine tumor of the duodenal bulb. Immunohistochemistry staining was positive for chromogranin-A, synaptophysin, caudal-type homeobox 2 (CDX2), and cytokeratin (CK) CAM 5.2, and the Ki-67 proliferative index was less than 3%. A CT scan and DOTATATE scan were then performed to further assess the mass and look for metastasis. There was no conclusive evidence of the mass or metastasis in either investigation. The patient was then scheduled for a laparoscopy-assisted robotic transduodenal resection of the tumor (Figure 1C). As the tumor could not be visualized during laparoscopy, an intraoperative endoscopy was performed to localize and transilluminate the tumor. The lesion was then identified laparoscopically, isolated, and removed via a robotic-guided transduodenal excision to minimize complications like bleeding and injury to neighboring organs. During the surgery, two peritumoral lymph nodes were noted to be enlarged and were also resected. The patient also underwent cholecystectomy during the procedure prophylactically in the event that the patient would require postoperative treatment with lanreotide in the future, as this could otherwise result in cholelithiasis [15]. At pathology, H&E staining showed the presence of neuroendocrine cells with fibrovascular septae in between (Figure 1D).

**Figure 2:**
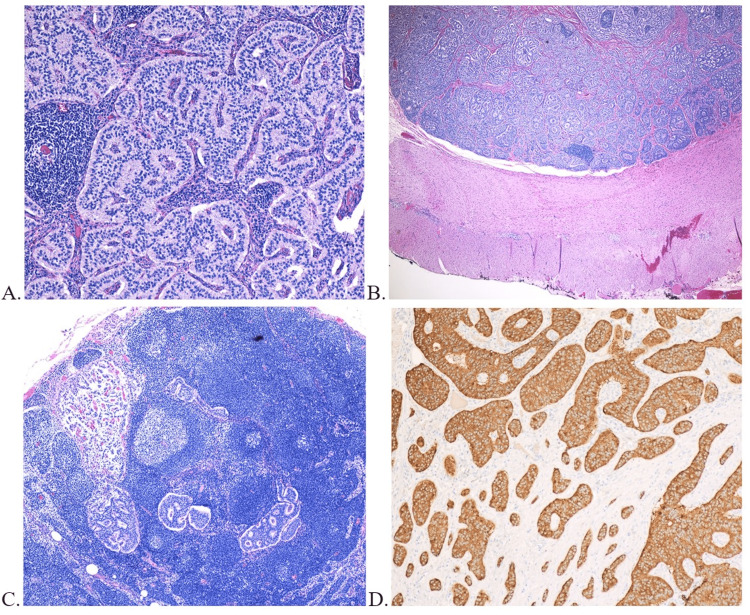
A) Histology slide showing nests of the neuroendocrine cells; B) Tumor invading the submucosa and extending up to muscularis propria; C) Histology slide of the lymph node being involved by the tumor; D) Immunohistochemical staining of the mass showing synaptophysin positivity of the neuroendocrine cells

At pathology, H&E staining showed nests of the neuroendocrine cells (Figure 2A).

The tumor was approximately 1 cm in size, invading the submucosa and extending up to, but not invading through, the muscularis propria (Figure 2B). Also, surgical resection margins were tumor-free. One of two periduodenal lymph nodes was positive for metastasis and measured 5 x 2.3 mm in dimension (Figure 2C). The final pathological stage was T1N1, with both the tumor (Figure 2D) and lymph node being positive for synaptophysin, corresponding to Stage 3b of TNM staging [12].

Since imaging was unable to confirm the diagnosis of the NET prior to the resection, further immunological markers were sent for the resected mass, of which carbonic anhydrase IX (CAIX) and paired-box gene 8 (PAX8) were positive. These additional markers are specific to well-differentiated neuroendocrine tumors and therefore helped us confirm our diagnosis of the well-differentiated NET.

During her hospital course, the patient was afebrile and hemodynamically stable, and her surgical scar was also healing well. On postoperative day two, a Gastrografin swallow study showed no leaks, and she was started on clear liquids. She was then transitioned to a soft-food diet the following day. Improvements were noted in both hemoglobin and hematocrit levels in the postoperative labs, with a rise in hemoglobin from 10.4 (Table 1) to 11.4 g/dL (Table 2) and hematocrit from 33.9% (Table 1) to 36.2% (Table 2).

**Table 1:** Laboratory results prior to diagnosis and resection of the NET

	Lab results	Reference
Hemoglobin	10.4 g/dL	12.1-15.1 g/dL
Hematocrit	33.9%	36%-48%
Mean corpuscular volume	89.4fl	80-100 fL
Ferritin	8ng/mL	10-291 ng/mL
Iron	47 mcg/dl	37-145 mcg/dl
Iron saturation (%)	12%	20%-55%
Total iron-binding capacity (TIBC)	394 mcg/dl	228-428 mcg/dl

**Table 2:** Laboratory results after the diagnosis and resection of the NET

	Lab results	Reference
Hemoglobin	11.4 g/dL	12.1-15.1 g/dL
Hematocrit	36.2%	36%-48%
Mean corpuscular volume	91.2fl	80-100 fL
Ferritin	15ng/mL	10-291 ng/mL
Iron	62 mcg/dl	37-145 mcg/dl
Iron saturation (%)	17%	20%-55%
Total iron-binding capacity (TIBC)	358 mcg/dl	228-428 mcg/dl

She had a BreathTek® test for *H. pylori* upon her follow-up visit, which was negative, and her epigastric discomfort had resolved.

Case two

**Figure 3:**
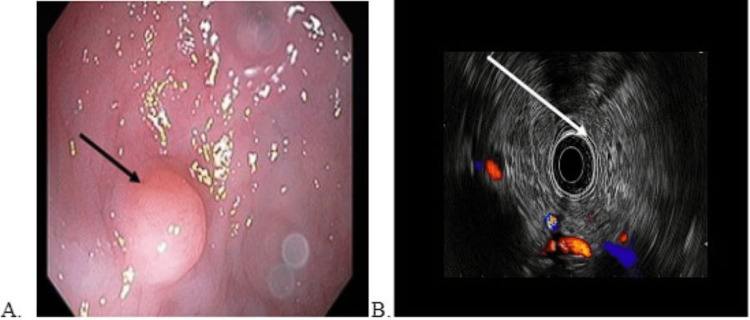
A) Colonoscopic visualization of the mass in the rectum; B) Endoscopic image of the lesion during EMR via EUS EMR: endoscopic mucosal resection; EUS: endoscopic ultrasound

A 51-year-old female came to the gastroenterology clinic with complaints of occasional constipation and rectal pain. She had no history of bleeding per rectum, weight loss, loss of appetite, or changes in bladder or bowel habits. She had a known history of hypertension and hypercholesterolemia, for which she took valsartan and simvastatin, respectively. The only surgery she reported having was a cesarean section. The patient underwent a colonoscopy, which showed a rectal polypoid lesion (Figure 3A), and jumbo biopsies showed a grade 1 well-differentiated neuroendocrine tumor.

Serum chromogranin-A levels were checked, which were elevated at 171 ng/mL. An EUS was done for staging, which revealed a hypoechoic mass in the rectum, 8 cm from the anal verge. It was well-defined and confined to the deep mucosa (layer 2), without any submucosal extension. The mass measured 9 mm in length and 6 mm in thickness (Figure 3B), corresponding with stage 1 of the AJCC TNM staging system [10].

**Figure 4:**
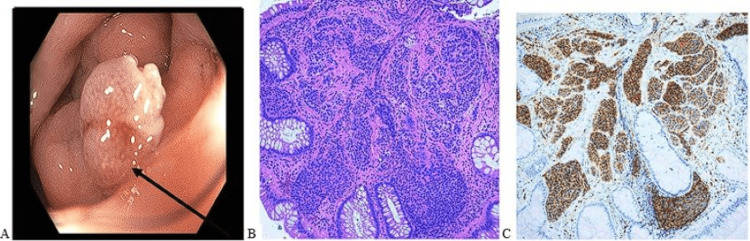
A) Mass being endoscopically resected by EMR; B) Histological slide of the resected mass, showing the presence of characteristic neuroendocrine cells; C) Immunohistochemical staining of the mass showing synaptophysin positivity of the neuroendocrine cells EMR: endoscopic mucosal resection

The lesion was then resected endoscopically via EMR (Figure 4A).

Pathology showed a 9 mm well-defined neuroendocrine tumor with islands of monotonous neuroendocrine cells with amphophilic granular cytoplasm and round nuclei with "salt and pepper" chromatin, minimal pleomorphism, no mitotic activity, and no necrosis (Figure 4B). The tumor cells were positive for synaptophysin (Figure 4C), CAM 5.2, and anticytokeratin monoclonal antibodies AE1/AE3; the Ki-67 proliferative index was < 1%. The resected margins were free of the tumor, giving pathological confirmation of stage 1. Post-resection, the patient was advised to consume a high-fiber diet and do abdominal exercises for weight loss and to promote bowel movements.

The patient recovered well after the procedure and was hemodynamically stable. Improvements were noted in both her hemoglobin and hematocrit levels in the postoperative labs, with a rise in hemoglobin from 11.7 (Table 3) to 13.2 g/dL (Table 4), and hematocrit from 37.2% (Table 3) to 40.8% (Table 4).

**Table 3:** Laboratory results prior to diagnosis and resection of the NET NET: neuroendocrine tumor

	Lab results	Reference
Hemoglobin	11.7 g/dL	12.1-15.1 g/dL
Hematocrit	37.2 %	36-48%
Mean corpuscular volume	84 fl	80-100 fL

**Table 4:** Laboratory results after the diagnosis and resection of the NET NET: neuroendocrine tumor

	Lab results	Reference
Hemoglobin	13.2 g/dL	12.1-15.1 g/dL
Hematocrit	40.8 %	36-48%
Mean corpuscular volume	84 fl	80-100 fL

Her symptoms of rectal pain resolved, and her serum chromogranin A levels normalized. She had a repeat colonoscopy one year later that showed no residual or recurrence of the tumor.

## Discussion

As GEP-NETs often present late with metastasis, incidental tumors should be promptly investigated through imaging studies and biopsies. When symptomatic, GEP-NETs share symptoms with many other gastrointestinal disorders, such as dyspepsia, abdominal pain or discomfort, and rectal pain. These symptoms are often overlooked and misdiagnosed as irritable bowel syndrome (IBS). Also, the majority of these tumors are non-functional, making diagnosis challenging.

Gastroenteropancreatic neuroendocrine tumors are now more commonly diagnosed as incidental findings in imaging studies. These include nonfunctional NETs, gangliocytic paragangliomas, gastrinomas, and rarely, poorly differentiated neuroendocrine carcinomas. Similar to other gastrointestinal NETs, duodenal and rectal NETs are also found incidentally on routine screening or endoscopic imaging done for the evaluation of non-specific symptoms. While imaging is useful for detecting the tumor, biopsy, and histopathology are commonly used to identify the type and grade of the tumor. During an upper endoscopic evaluation, there are clear indications for biopsy, such as suspicion of neoplasia, abnormal-appearing mucosa with suspected inflammation, and whether a biopsy of normal-appearing or inflamed mucosa is likely to contribute to patient management in upper gastrointestinal disease [16]. The best course of treatment for NETs depends on the tumor's size, location, histological grade, stage, and type [17].

Since GEP-NETs usually have a moderate growth rate, tumor markers, and imaging should be performed at regular intervals (four to 12 months). Changes in tumor markers should not be the only factor considered when choosing a treatment, as functional imaging can be helpful in monitoring patients with NETs. Long-term follow-ups (five to 10 years) are recommended for small bowel and colorectal NETs, as there is a risk of potential recurrence many years after the initial diagnosis. Depending on the biological aggressiveness of the neoplasm, determined by the Ki-67 index, mitotic activity, the degree of differentiation, the presence of lymphovascular or perineural invasion, marginal elimination, and the quality of the surgery performed, radical postoperative monitoring via imaging studies is carried out for resected GEP-NETs and can be conducted at intervals of six months to two years [18].

In our first case, the neuroendocrine tumor appeared as a benign polypoid submucosal lesion during her upper GI endoscopy. The first biopsy revealed only the presence of hyperplastic glands. Only after further testing with EUS-guided FNA/FNB did pathological examination reveal the presence of a neuroendocrine tumor. The tumor was then resected using a robotic approach, as robotic surgery is a minimally invasive surgical technique that uses robotic arms controlled by a surgeon to perform precise movements with enhanced dexterity and visualization. Robotic surgery offers several potential advantages over traditional open surgery, including smaller incisions, reduced blood loss, shorter hospital stays, faster recovery times, and fewer periprocedural complications. As the tumor, which was TNM stage 3b, was resected along with the affected periduodenal lymph node, the five-year prognosis of the patient improved from 91% to 100% [13], rather than deteriorating to a higher stage with a poorer prognosis. Our second case was a patient who underwent a colonoscopy for altered bowel habits and occasional rectal pain, which showed a rectal polypoid lesion, and a biopsy revealed a well-differentiated NET. The EUS showed a <1 cm stage 1 deep mucosal tumor that was also resected by a minimally invasive procedure called endoscopic mucosal resection (EMR).

In both cases, patients originally presented to our clinic with anemia that improved after the removal of their respective lesions. Anemia associated with NETs can be due to iron deficiency, which can be attributed to microscopic bleeding associated with mucosal involvement or deeper invasion within the GI epithelium. Anemia in NET cases can also be due to anemia of chronic disease, as NETs produce proinflammatory cytokines such as interleukin-6 (IL-6) and tumor necrosis factor-alpha (TNF-α), which decrease erythropoietin production, resulting in reduced protection of mature erythrocytes and a reduced rate of bone marrow erythropoiesis. Interleukin-6 is also a modulator of hepcidin, which, when induced, blocks iron absorption in the duodenum and iron release from macrophages [19]. This helps explain why the removal of these lesions resulted in an improvement in both the patients' hemoglobin and hematocrit levels.

This series highlights the need to further evaluate incidental, benign-looking tumors to better understand the causative etiology that produces vague symptoms in certain patients. Since NETs can remain dormant until advanced progression, these cases highlight the importance of early diagnosis and management and emphasize the importance for physicians to include them in their differential diagnoses and to consider further investigation of suspicious masses seen in endoscopic studies.

In accordance with the National Comprehensive Cancer Network® (NCCN®) 2022 guidelines, patients should be evaluated three to 12 months after resection and subsequently every six to 12 months for a total of 10 years. Multi-phase CTs or MRIs are recommended as the first imaging test for patients who may have a distant metastatic illness. The patients' 24-hour urine or plasma 5-hydroxy indoleacetic acid (5-HIAA) can be assessed to monitor them and track the course of their disease [20]. Both our patients are under surveillance and are disease-free thus far.

## Conclusions

Gastrointestinal NETs are a common digestive tract disorder whose diagnostic frequency has increased in recent years due to advancements in imaging methods and technology. The increased incidence also supports the need for advanced diagnostic modalities and early interventions for definitive management. Increased reporting of these tumors and periodic updates to the literature on NETs can improve clinicians’ knowledge of neuroendocrine tumors. Neuroendocrine tumors have to be part of the early differentials in patients who present with multisystem symptoms and vague masses seen in imaging studies. Further research is needed on early intervention with new and improved pharmacotherapeutic agents and the benefits of minimally invasive and robot-assisted surgical interventions as methods of treating NETs. Early-stage diagnosis is critical to prevent metastases and improve survival outcomes.
